# Genome-Wide Identification of the *ZjWPR* Gene Family in Chinese Jujube Provides Functional Insights into Its Response to Jujube Witches’ Broom

**DOI:** 10.3390/plants15132094

**Published:** 2026-07-06

**Authors:** Pan Li, Caihua Xing, Jiaqi Sun, Yunjie Wang, Kunyi Lv, Enshun Jiang, Shoule Wang, Zhongtang Wang, Changfeng Ai, Xueqing Yan, Xuan Zhao, Qiong Zhang

**Affiliations:** 1 Shandong Academy of Agricultural Sciences, Jinan 250100, China; lipansaas@163.com (P.L.); xingcaihualy@163.com (C.X.); enshunjiang@163.com (E.J.); wangshoule17@mails.ucas.ac.cn (S.W.); sdgss213@163.com (Z.W.); 2 College of Horticulture, Hebei Agricultural University, Baoding 071001, China; 18733589812@163.com (J.S.); 15100266806@163.com (K.L.); 15264933693@163.com (C.A.); 17661855056@163.com (X.Y.); 3 Handan Academy of Agricultural Sciences, Handan 056001, China; wang15028030219@163.com

**Keywords:** Chinese jujube, *ZjWPR*, gene family, jujube witches’ broom, leaf chlorosis

## Abstract

*WPR* (*WEB1/PMI2-related*) genes play a crucial role in regulating chloroplast movement and leaf coloration in plants. Previous studies have shown that these genes are implicated in leaf yellowing, both in *Arabidopsis thaliana* and in *Paulownia fortunei* following infection with *Paulownia* witches’ broom. To investigate the functions of the *ZjWPR* genes in jujube, bioinformatics methods were employed to identify the *ZjWPR* gene family in jujube, analyze their protein physicochemical properties, gene structure, evolutionary relationships, and cis-acting elements in this study. The results revealed that the *ZjWPR* gene family in jujube comprised 10 members. Phylogenetic analysis showed that *WPR* genes were divided into two classes, with *ZjWPR* genes distributed across three subgroups within Class II. Conserved motif analysis indicated that motif 2, motif 3, motif 7, and motif 8 were the most highly conserved and most genes exhibited similar structures. Cis-element analysis in their promoter suggested that *ZjWPR* genes were regulated by multiple hormones and were associated with stress responses such as low temperature and drought. Moreover, all *ZjWPR* genes contained light-responsive elements. Expression analysis of the *ZjWPR* gene family under Jujube Witches’ Broom (JWB) stress showed that *ZjWPR4* and *ZjWPR5* were significantly up-regulated in JWB-susceptible jujube cultivars following phytoplasma infection, whereas no significant changes were detected in JWB-resistant cultivars. Additionally, the expression levels of *ZjWPR2*, *ZjWPR3*, and *ZjWPR6* were also altered in response to infection, suggesting their potential involvement in the response to JWB stress and the associated leaf chlorosis process. Moreover, transient overexpression of *ZjWPR4* and *ZjWPR5* in sour jujube leaves led to significant reductions, in critical photosynthetic parameters, including Fv/Fm, Fq′/Fm′, and ETR compared with WT, thereby reinforcing their functional contribution to JWB-associated leaf yellowing. This study provides valuable insights for further functional characterization of the *ZjWPR* gene family in mediating JWB-induced leaf yellowing and related metabolic pathways.

## 1. Introduction

The *WPR* (*WEB1/PMI2-related*) gene family is known to play critical roles in regulating chloroplast movement and leaf coloration in plants. The family was first identified through studies on chloroplast photorelocation movement-deficient mutants of *Arabidopsis thaliana*. Initial studies classified the family into two subfamilies: *PMI2* (*PLASTID MOVEMENT IMPAIRED 2*) and *WEB1* (*WEAK CHLOROPLAST MOVEMENT UNDER BLUE LIGHT 1*). Mutations in *PMI2* impair chloroplast movement across a range of light intensities, whereas mutations in *WEB1* primarily affect chloroplast motility under blue light conditions [1,2,3,4]. Subsequent research, which revealed that *Arabidopsis* contains a total of 14 *WPR* members, led to a refined phylogenetic classification into four distinct subfamilies: *WEB1*, comprising 5 members (e.g., *AtWPR1*), which are primarily involved in chloroplast accumulation under weak blue light, *PMI2*, with 3 members (e.g., *AtWPR11*), mainly associated with the avoidance response under strong blue light, *WPRa*, containing 3 members, and *WPRb*, also containing 3 members. While the functions of *WPRa* and *WPRb* remain less characterized, potentially indicating functional redundancy or specialization, it is established that WPR proteins act as scaffold proteins localized to the outer chloroplast membrane. They interact with photoreceptors (e.g., phototropins) and cytoskeletal components (e.g., actin), forming a complex signaling network that translates light signals into mechanical forces driving chloroplast movement [5,6].

Current research shows that most *WPR* genes contain a highly conserved WEMBL (Weak Chloroplast Movement under Blue Light) domain, which facilitates protein–protein interactions. Evidence indicates that the *WPR* family is closely associated with plant photorelocation responses, particularly through the formation of a WEB1–PMI2 complex that regulates actin filament dynamics during light avoidance [2,6,7]. Studies have also reported that the *WPR* family is involved in leaf yellowing processes. For instance, various classes of mutants defective in chloroplast avoidance movement exhibit increased susceptibility to high-light damage compared to wild-type plants. In these mutants, damage to the photosynthetic apparatus occurs more rapidly under high-light conditions, leading to accelerated leaf bleaching and necrosis [8]. This link between *WPR* function, photoprotection, and leaf coloration suggests a potential role for *WPR* genes in other types of leaf color changes, such as those caused by pathogen stress.

Subsequent investigations have increasingly highlighted the importance of the *WPR* gene family across plant species. For example, 23 *WPR* members have been identified in apple, 5 in grape, 9 in *Populus trichocarpa*, and 16 in *Paulownia fortunei* [1,9]. These findings indicate significant variation in *WPR* gene family size across species. However, no studies have yet reported *WPR* genes in *Ziziphus jujuba* Mill., a member of the Rhamnaceae family.

*Ziziphus jujuba* Mill., which is native to China, has been cultivated for nearly 7000 years and holds considerable economic and nutritional value [10,11,12]. Jujube witches’ broom (JWB), caused by phytoplasma infection, is one of the most devastating diseases affecting jujube production. It is highly contagious, difficult to manage, and often leads to plant death within 2–3 years [13]. In recent years, JWB has spread rapidly across multiple provinces in China, posing a major threat to the sustainability of the jujube industry. Typical symptoms of JWB include leaf chlorosis, phyllody, witches’ broom-like branching, and eventual plant death. Chlorotic leaves are typically narrow, emerald green, and exhibit prominent veins. Following flowering, leaves gradually yellow, curl at the margins, become brittle, lose luster, and may eventually turn brown and fall off [14,15]. Previous studies have shown that JWB phytoplasma infection downregulates genes involved in chlorophyll biosynthesis, leading to reduced chlorophyll content. However, whether *WPR* genes are involved in this chlorosis remains to be elucidated.

In this study, the newly assembled T2T genome of Dongzao jujube was utilized to comprehensively identify members of the *ZjWPR* gene family in jujube. A systematic bioinformatics analysis was conducted, and transcriptomic data from resistant and susceptible jujube cultivars under JWB phytoplasma stress were integrated to screen for *ZjWPR* genes potentially responsive to pathogen infection. It revealed that the expression of *ZjWPR4* and *ZjWPR5* was significantly up-regulated in JWB-susceptible jujube cultivars following phytoplasma infection, whereas no significant changes were detected in JWB-resistant cultivars. Additionally, the expression levels of *ZjWPR2*, *ZjWPR3*, and *ZjWPR6* were also altered in response to infection. These findings suggested that *ZjWPR2*, *ZjWPR3*, *ZjWPR4*, *ZjWPR5*, and *ZjWPR6* may play regulatory roles in the development of JWB-associated leaf yellowing. This study provides preliminary insights into the role of the *ZjWPR* gene family in JWB-induced leaf chlorosis, laying a foundation for future investigations into its regulatory mechanisms.

## 2. Results

### 2.1. Identification of the ZjWPR Gene Family

Using a combined approach of homology sequence comparison and conserved domain analysis, ten *WPR* genes were identified in the jujube genome and designated as *ZjWPR1* to *ZjWPR10*. Further physicochemical characterization analysis of the *ZjWPR* gene family, including amino acid numbers, molecular weight, atomic composition, isoelectric point, instability index, aliphatic amino acid index, and hydrophilicity, was conducted. The amino acid numbers of the encoded proteins ranged from 213 to 898 residues, with ZjWPR1 being the largest (898 aa) and ZjWPR9 the smallest (213 aa). The molecular weights varied from 23,989.32 to 98,162.54 Da, with ZjWPR1 exhibiting the highest and ZjWPR9 the lowest molecular weight. The isoelectric points ranged from 5.03 to 9.71, with ZjWPR10 showing the highest value and ZjWPR7 the lowest. Instability index values ranged from 42.08 to 57.76, indicating that all members are potentially unstable proteins (Instability index > 40). The aliphatic amino acid index ranged from 76.57 to 86.45, with ZjWPR7 having the highest and ZjWPR10 the lowest. Hydrophilicity values ranged from −0.98 to −0.664. These physicochemical analyses suggest functional diversity among the members of the *ZjWPR* gene family (Table 1).

### 2.2. Pseudo-Chromosome Localization of ZjWPR Gene Family Members

**Figure 1:**
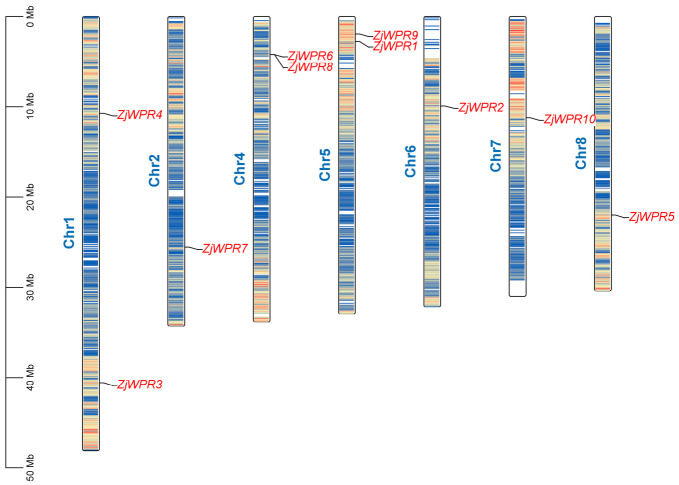
Location of pseudochromosomes of the *ZjWPR* gene family members. All identified *ZjWPR* members are derived from the nuclear genome.

Chromosomal localization analysis revealed that the 10 *ZjWPR* genes were distributed across seven pseudochromosomes. Specifically, *ZjWPR3* and *ZjWPR4* were located in distinct regions of chromosome 1, *ZjWPR6* and *ZjWPR8* are located in close proximity on chromosome 4 and may be arranged in tandem, and *ZjWPR1* and *ZjWPR9* were located in different regions of chromosome 5 (Figure 1).

### 2.3. Phylogenetic Analysis of the ZjWPR Gene Family

**Figure 2:**
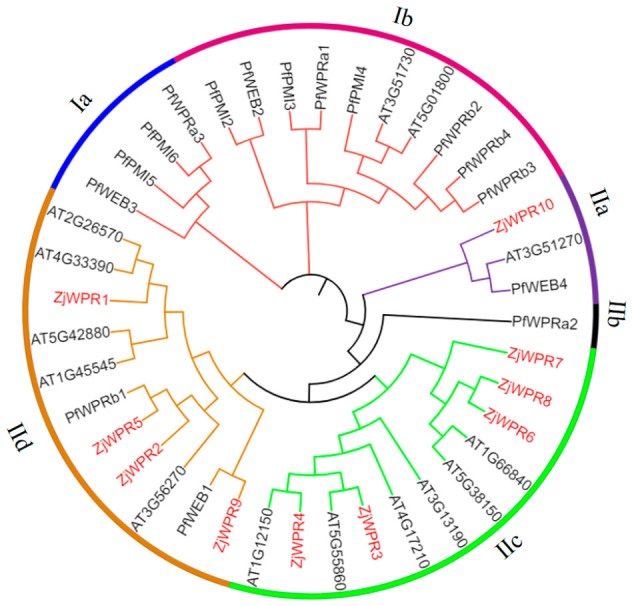
Phylogenetic evolutionary tree of the *WPR* gene family in jujube, *Arabidopsis thaliana* and *Paulownia fortunei*.

To explore the evolutionary relationships of the *ZjWPR* gene family in jujube, a phylogenetic tree was constructed using *WPR* gene sequences from jujube, *Arabidopsis thaliana*, and *Paulownia fortunei* (Seem.) Hemsl. (Figure 2). The analysis revealed two major clades (Group I and Group II), with Group I containing two subgroups, Ia and Ib, and Group II containing four subgroups, IIa~IId. *ZjWPR* genes were distributed across three subgroups within Group II: IIa, IIc, and IId. Group IIa contained only *ZjWPR10*, Group IIc included *ZjWPR3*, *ZjWPR4*, *ZjWPR6*, *ZjWPR7*, and *ZjWPR8*, and Group IId contained *ZjWPR1*, *ZjWPR2*, *ZjWPR5*, and *ZjWPR9*. Notably, *ZjWPR10* clustered with *PfWEB4* of *Paulownia*, ZjWPR9 was closely related to *PfWEB1*, and *ZjWPR2* and *ZjWPR5* were closely associated with *PfWPRb1*. These results suggested that members of the *ZjWPR* gene family in jujube may share similar functions with some members of the *Paulownia fortunei PfWPR* gene family.

### 2.4. Conservation Motifs, Conserved Domains and Structural Analysis of the ZjWPR Gene Family

**Figure 3:**
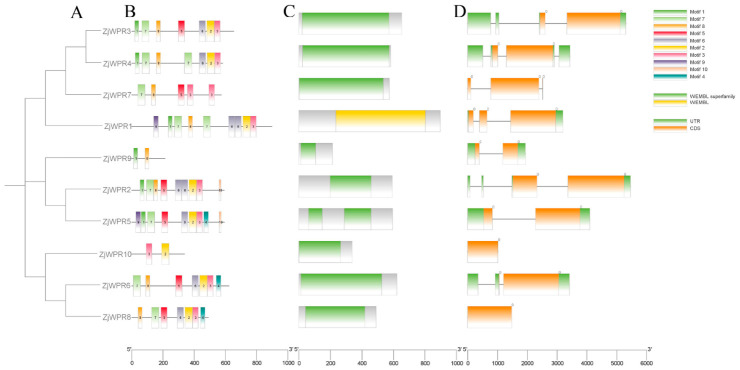
Analysis of conserved motifs and gene structure of the *ZjWPR* gene family. (**A**) Phylogenetic tree analysis, (**B**) Conservation motif analysis, (**C**) Conservation domain analysis, (**D**) Gene structure analysis.

To further investigate the characteristics of the *ZjWPR* gene family in jujube, we performed conserved motif analysis on its members (Figure 3). The results revealed the presence of ten conserved motifs in the *ZjWPR* family. Each gene contained between 2 and 9 motifs, and *ZjWPR6* and *ZjWPR8* shared seven identical motifs. Motif 9 was exclusively presented in *ZjWPR1* and *ZjWPR5*, while motif 10 was only found in *ZjWPR2* and *ZjWPR5*. Among these, the vast majority of genes contain motif 2, motif 3, motif 7, and motif 8 (Figure 3B), suggesting that these four conserved motifs may represent the fundamental motifs of the *ZjWPR* gene family.

Subsequently, conserved domain analysis of the *ZjWPR* family members revealed that only *ZjWPR1* contained a highly conserved WEMBL domain, while the other nine members all belonged to the WEMBL superfamily (Figure 3C). This indicated that all *ZjWPR* family members in jujube were involved in regulating the avoidance movement of chloroplast actin filaments in response to light. Furthermore, analysis of the gene structures of the *ZjWPR* family members (Figure 3D) showed that *ZjWPR1* and *ZjWPR7* contained two introns, *ZjWPR2*, *ZjWPR3*, *ZjWPR4*, *ZjWPR5, ZjWPR6* and *ZjWPR9* possessed one intron, and *ZjWPR8* and *ZjWPR10* had no introns. This suggested that *ZjWPR1*, *ZjWPR7, ZjWPR2*, *ZjWPR3*, *ZjWPR4*, *ZjWPR5*, *ZjWPR6* and *ZjWPR9* may undergo alternative splicing to produce different transcripts, potentially enabling adaptation to environmental changes, whereas *ZjWPR8*/*10* lacked the capacity for alternative splicing.

### 2.5. Analysis of Cis-Regulatory Elements in the Promoter of ZjWPR Gene Family

**Figure 4:**
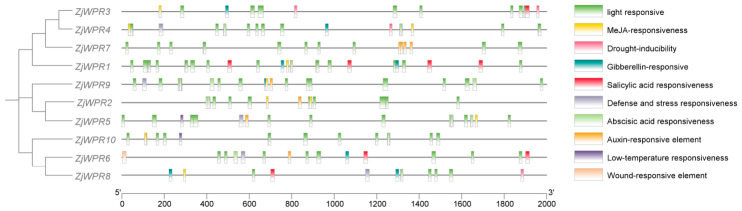
Cis-regulatory element analysis of *ZjWPR* Promoters.

To further elucidate the functions of the *ZjWPR* gene family, we performed cis-acting element analysis on its members. The results revealed the presence of ten major types of regulatory elements in the promoter regions of *ZjWPR* genes. Among these, five are phytohormone-responsive cis-acting elements: jasmonic acid-responsive, abscisic acid-responsive, auxin-responsive, gibberellin-responsive, and salicylic acid-responsive elements, indicating that the expression of *ZjWPR* genes is regulated by multiple plant hormones. Additionally, other regulatory elements include light-responsive, wound-responsive, defense and stress-responsive, drought-inducible, and low-temperature-responsive elements, suggesting the involvement of *ZjWPR* genes in processes such as photosynthesis, as well as adversity stress responses like drought and cold tolerance (Figure 4).

### 2.6. Expression Analysis of the ZjWPR Gene Family in Jujube in Response to JWB Stress

**Figure 5:**
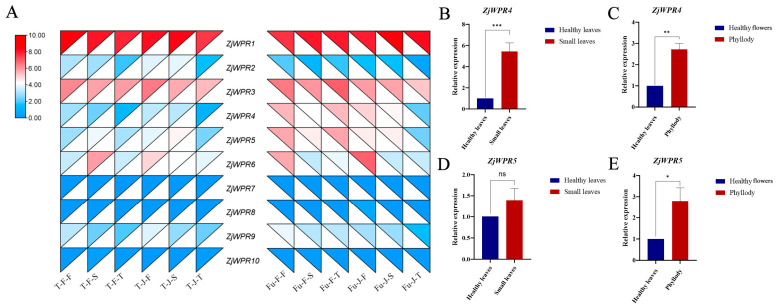
Expression analysis of the ZjWPR gene family in jujube in response to JWB stress. (**A**) The heatmap shows the expression pattern of ZjWPR gene family members in disease-resistant cultivar ‘T13’ and susceptible cultivar ‘Fuxiang’ in response to JWB stress; (**B**,**D**), Relative expression of *ZjWPR4* and *ZjWPR5* in healthy leaves and small leaves induced by JWB in susceptible cultivar ‘Pozao’; (**C**,**E**), Relative expression of *ZjWPR4* and *ZjWPR5* in healthy leaves and phyllody leaves induced by JWB in susceptible cultivar ‘Pozao’. Experiments were conducted in three biological replicates. Data are presented as mean ± SD and analyzed via Student’s *t*-test (* *p* < 0.05, ** *p* < 0.01, *** *p* < 0.001, ns = non-significant).

In order to reveal whether *ZjWPR* gene family responds to the stress caused by jujube witches’ broom, expression analysis of the *ZjWPR* genes in jujube was performed using leaf samples collected from the jujube witches’ broom disease-resistant cultivar ‘T13’ and susceptible cultivar ‘Fuxiang’, which were grafted onto either healthy or infected rootstocks under field conditions. Leaf samples were collected at three time points. The first sampling was performed when the susceptible cultivar ‘Fuxiang’, grafted onto infected rootstocks, exhibited typical jujube witches’ broom symptoms (e.g., reduced leaf size and severe branch deformation). Leaf samples from cultivar ‘T13’ were collected over the same period. Subsequent samples were taken at 15-day intervals. As shown in Figure 5, the heatmap showed that compared with the materials grafted onto healthy rootstocks, the expression levels of *ZjWPR4* and *ZjWPR5* significantly increased in the susceptible cultivar ‘Fuxiang’ grafted onto infected rootstocks, while no notable change was observed in the resistant cultivar ‘T13’ before and after stress (Figure 5A). The differential expression of *ZjWPR4* and *ZjWPR5* between resistant and susceptible cultivars suggested that these genes respond to JWB stress and may be involved in the JWB-induced leaf yellowing. Furthermore, in field-grown susceptible cultivar ‘Pozao’, the expression level of *ZjWPR4* was significantly elevated in JWB-induced small leaves compared to healthy leaves, and *ZjWPR5* expression also increased, though not significantly (Figure 5B,D). In JWB-induced phyllody leaves, both *ZjWPR4* and *ZjWPR5* expression levels were significantly higher compared to those in healthy leaves (Figure 5C,E). Additionally, the expression of *ZjWPR6* was significantly upregulated at the S2 stage in the resistant cultivar ‘T13’ grafted onto infected rootstocks compared with those grafted onto healthy rootstocks (Figure 5A). Furthermore, in the susceptible cultivar ‘Fuxiang’ grafted onto infected rootstocks, the expression of *ZjWPR2*, *ZjWPR3*, *ZjWPR4*, *ZjWPR5* and *ZjWPR6* all showed an initial decrease followed by a recovery pattern across all three development stages compared with control (Figure 5A). These expression patterns indicated that these genes were responsive to JWB stress and may be associated with leaf yellowing induced by JWB.

**Figure 6:**
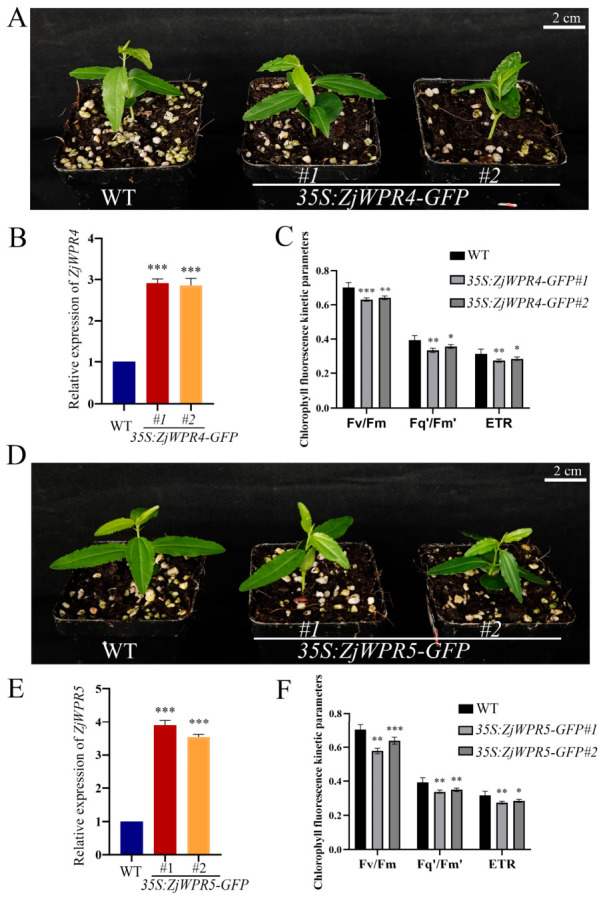
Function validation of *ZjWPR4* and *ZjWPR5* in sour jujube. (**A**) Phenotypic images of WT and *35S:ZjWPR4-GFP* overexpressing lines of sour jujube. (**B**) Relative expression of *ZjWPR4* in WT and *35S:ZjWPR4-GFP* overexpressing lines of sour jujube. (**C**) Chlorophyll fluorescence kinetic parameters in WT and *35S:ZjWPR4-GFP* overexpressing lines of sour jujube. (**D**) Phenotypic images of WT and *35S:ZjWPR5-GFP* overexpressing lines of sour jujube. (**E**) Relative expression of *ZjWPR5* in WT and *35S:ZjWPR5-GFP* overexpressing lines of sour jujube. (**F**) Chlorophyll fluorescence kinetic parameters in WT and *35S:ZjWPR5-GFP* overexpressing lines of sour jujube. Experiments were conducted in three biological replicates. Data are presented as mean ± SD and analyzed via Student’s *t*-test (* *p* < 0.05, ** *p* < 0.01, *** *p* < 0.001).

To further investigate the functional roles of *ZjWPR4* and *ZjWPR5*, we transiently overexpressed each gene in sour jujube leaves under the control of the *CaMV 35S* promoter (Figure 6A,B,D,E). Chlorophyll fluorescence kinetic analysis revealed that, relative to wild-type (WT) controls, the *35S:ZjWPR4-GFP* and *35S:ZjWPR5-GFP* lines exhibited significant reductions in key photosynthetic parameters, including Fv/Fm (maximum quantum yield of PSII), Fq′/Fm′ (light-adapted effective quantum yield of PSII), and ETR (electron transport rate) (Figure 6C,F). These impairments indicated compromised photochemical efficiency and diminished capacity for linear electron transport, consistent with systemic disruption of photosystem II functionality. Critically, the observed declines in photosynthetic performance preceded visible leaf chlorosis, raising the possibility that *ZjWPR4* and *ZjWPR5* overexpression is mechanistically associated with JWB-associated yellowing, not merely as a correlative marker but as a potential driver of photosynthetic deterioration. Collectively, these findings suggested a potential link between JWB-induced upregulation of *ZjWPR4* and *ZjWPR5* and the development of leaf yellowing symptoms.

## 3. Discussion

Members of the *WPR* family have been reported to induce leaf yellowing in *Arabidopsis thaliana* [8,16]. Studies indicated that elevated expression levels of *AtWEB1* and *AtPMI2* in *Arabidopsis* were associated with leaf yellowing phenotypes. Following infection with *Paulownia* witches’ broom phytoplasma, the expression of the *WPR* gene family underwent significant changes [9,17]. Specifically, *PfWEB3*, *PfWPRb2*, and *PfWPRb3* were markedly up-regulated in infected plants, suggesting that these genes may cooperatively regulate the leaf yellowing process in *Paulownia* witches’ broom [9,18]. However, the role of the *ZjWPR* gene family in jujube witches’ broom induced leaf yellowing remains unclear.

In this study, a total of 10 *WPR* family members were identified in the jujube genome, designated as *ZjWPR1* to *ZjWPR10*. Comparative genomic analysis revealed considerable variation in the number of *WPR* members among different species—for example, 23 in apple, 5 in grape, 9 in *Populus trichocarpa*, 14 in *Arabidopsis thaliana*, and 16 in *Paulownia fortunei* [1,9]—suggesting functional diversification of this family across species. Phylogenetic analysis classified the *WPR* family genes into two major groups, with all *ZjWPR* genes falling into Group II, indicating their potential functional similarity. Further evolutionary analysis showed high homology between *ZjWPR* genes and their orthologs in *Paulownia fortunei*. Conserved motif analysis demonstrated that most *ZjWPR* members shared similar gene structures, and all contained highly conserved motifs (motif2, motif3, motif7, motif8), implying possible functional commonalities. Promoter analysis indicated that *ZjWPR* genes contained various phytohormone-responsive cis-elements as well as elements related to light, low temperature, and drought stress, suggesting their roles in photosynthesis, drought response, and cold stress adaptation. These findings provide important clues for further functional characterization of *ZjWPR* genes.

Jujube witches’ broom (JWB) is a highly infectious and severe phytoplasma-induced disease, with leaf yellowing being one of its most characteristic symptoms [14,15,19,20]. However, the underlying molecular mechanisms driving this symptom remain largely unclear. In this study, we found that *ZjWPR4* and *ZjWPR5* were significantly upregulated in the susceptible jujube cultivar ‘Fuxiang’ upon infection with JWB, whereas their expression remained unchanged in the resistant cultivar ‘T13’. In the susceptible cultivar ‘Pozao’, when compared with healthy leaves, both genes exhibited markedly higher expression levels in JWB-infected symptomatic leaves, including small leaves and phyllody leaves. Moreover, transient overexpression of *ZjWPR4* or *ZjWPR5* in sour jujube leaves resulted in a pronounced decline in photosynthetic performance. Collectively, these results suggest that JWB phytoplasma might promote leaf yellowing not merely as a passive consequence of infection, but actively by inducing aberrantly high expression of *ZjWPR4* and *ZjWPR5*, thereby disrupting host photosynthetic function.

Leaf yellowing is a hallmark symptom of JWB disease and is mechanistically linked to chlorophyll degradation and structural/functional impairment of the photosynthetic apparatus [21,22]. Our transient overexpression experiments provide direct functional evidence that elevated *ZjWPR4* and *ZjWPR5* expression causally impairs photosynthesis. Relative to WT controls, *35S: ZjWPR4-GFP* and *35S: ZjWPR5-GFP* lines showed significant reductions in key chlorophyll fluorescence parameters: Fv/Fm, Fq′/Fm′, and ETR. A decline in Fv/Fm reflects compromised photochemical efficiency of PSII, a well-documented indicator of photoinhibitory stress and chloroplast dysfunction under diverse biotic and abiotic stresses [23]. Reduced ETR signifies inhibition of linear electron transport, which likely diminishes ATP and NADPH production, thereby exacerbating metabolic imbalances in infected leaves [23]. Together, these physiological deficits raise the possibility that *ZjWPR4* and *ZjWPR5* overexpression is mechanistically associated with JWB-associated yellowing. In future work, examining whether overexpression of *ZjWPR4* and *ZjWPR5* leads to a reduction in leaf chlorophyll content may provide further evidence that the expression changes in *ZjWPR4* and *ZjWPR5* under JWB stress serve not merely as a correlative marker but as a potential driver of photosynthetic deterioration.

Although prior studies have associated *WPR* gene dysregulation with photosynthetic damage [8], the precise molecular mechanisms linking pathogen-induced *ZjWPR4* and *ZjWPR5* upregulation to PSII dysfunction remain poorly defined. WPR proteins are canonical scaffold proteins localized to the outer chloroplast envelope, where they physically couple photoreceptors (e.g., phototropins) to actin filaments to regulate light-directed chloroplast movement [5,6]. Given their robust induction specifically in JWB-infected leaves and the absence of comparable upregulation in resistant genotypes, we propose a stress-specific, non-canonical function: excessive accumulation of *ZjWPR4* and *ZjWPR5* may perturb chloroplast homeostasis, potentially via pathological remodeling of the actin cytoskeleton, leading to downstream repression of nuclear- and plastid-encoded photosynthesis-related genes. Such transcriptional reprogramming could directly impair PSII assembly, stability, or repair. Furthermore, ZjWPR4 and ZjWPR5 may interact with unidentified regulatory partners, including transcription factors or signaling kinases to indirectly compromise PSII function. These hypotheses represent testable frameworks for future mechanistic studies.

## 4. Materials and Methods

### 4.1. Materials

In this study, the susceptible Chinese jujube cultivar ‘Fuxiang’ (referred to as ‘Fu’) to jujube witches’ broom disease and the resistant cultivar ‘T13’, both cultivated at the Fuping Experimental Station of Hebei Agricultural University, were selected as plant materials. Scions from these two healthy cultivars were grafted onto either diseased or healthy rootstocks, respectively [24], and the resulting combinations were designated as Fu-F, T-F (F denotes infected rootstock), Fu-J, and T-J (J denotes healthy rootstock). All experimental trees were grown under natural conditions. After grafting, the rootstocks began to sprout, develop leaves, and grow gradually. Leaf samples were collected once typical jujube witches’ broom symptoms were observed, including small leaf size and severe branch deformation. Sampling was conducted every 15 days for a total of three sampling periods. Each sample was collected from three different trees. The first sampling was designated as the First period (F), followed by the Second (S) and Third (T) periods. Collected samples were immediately placed in an icebox, rapidly frozen in liquid nitrogen upon arrival in the laboratory, and stored at −80 °C for subsequent RNA extraction, cDNA synthesis, and further analysis.

For RT-qPCR analysis of *ZjWPR4* and *ZjWPR5* expression, leaf samples were collected from field-grown susceptible ‘Pozao’ jujube trees exhibiting jujube witches’ broom symptoms, including witches’ broom-induced small leaves and phyllody. Leaves from healthy ‘Pozao’ trees were used as controls. Total RNA was extracted from all samples, reverse-transcribed into cDNA, and subjected to quantitative PCR. Three biological replicates were performed to detect the expression levels of *ZjWPR4* and *ZjWPR5*.

### 4.2. Identification of the ZjWPR Gene Family in Jujube

The WPR protein sequence from *Arabidopsis* was used as a query to perform BLASTP analysis against the jujube genome database (https://www.ncbi.nlm.nih.gov/assembly/GCF_000826755.1 (accessed on 23 June 2026)) (URL 12 March 2023), aiming to identify members of the ZjWPR gene family in Chinese jujube (*Ziziphus jujuba* Mill.) [25]. Chromosomal locations and amino acid sequences of the identified genes were obtained from the NCBI (National Center for Biotechnology Information) database. Physicochemical properties, including isoelectric point and molecular weight, were analyzed using the online ProtParam tool (https://web.expasy.org/protparam/, accessed on 23 June 2026). Chromosomal distribution of the *ZjWPR* genes was visualized using TBtools software (version 2.441). Chromosomal localization maps can be generated using the “Gene Location Visualization” function in TBtools by submitting the corresponding GFF/GTF files.

### 4.3. Phylogenetic Analysis of the ZjWPR Gene Family in Jujube

WPR protein sequences from *Arabidopsis* and *Paulownia fortunei* were retrieved from the NCBI database. Multiple sequence alignment of WPR proteins from jujube, *Arabidopsis*, and *Paulownia fortunei* was conducted using the ClustalW algorithm in MEGA v7.0. A phylogenetic tree was then constructed using the neighbor-joining method to infer evolutionary relationships among the *WPR* genes.

### 4.4. Analysis of ZjWPR Gene Structure and Conserved Motifs

The gene structure of *ZjWPR* members was analyzed using TBtools software [26]. Conserved domains within the candidate genes were identified using the Conserved Domain Database (CDD) [27]. Additionally, conserved motifs were predicted using the MEME (Multiple Em for Motif Elicitation) suite (version 5.5.9) [28], and the results were visualized using TBtools. Gene structure, domain, and conserved motif diagrams were generated using the “Gene Structure Visualization” function in TBtools, with the corresponding files submitted accordingly.

### 4.5. Analysis of Cis-Acting Regulatory Elements in the ZjWPR Gene Promoters

The 2000 bp upstream promoter regions of *ZjWPR* genes were extracted using TBtools software. Potential cis-acting regulatory elements within these promoter sequences were identified using the PlantCARE database [29]. The identified elements were visualized using TBtools software for further analysis. Cis-acting element diagrams can also be displayed using the “Biological Sequence Visualization” function in TBtools after importing the corresponding files.

### 4.6. Transcriptome Analysis

Raw paired-end sequencing reads were processed using SeqPrep (version 1.3.2) and Sickle software (version 1.33) with default parameters to remove adapter sequences and low-quality reads. The cleaned reads were then aligned to the jujube reference genome (downloaded from NCBI) using TopHat (version 2.0.0) [30]. Gene expression levels were quantified as transcripts per million (TPM) using RSEM software (version 1.3.3) [31]. Transcriptome heatmaps can be produced using the “Heatmap Visualization” function in TBtools by submitting the transcriptome data.

### 4.7. Transient Overexpression of ZjWPR4 and ZjWPR5 in Sour Jujube

Sour jujube cotyledons were injected with *Agrobacterium tumefaciens* strain GV3101 harboring either the *35S:ZjWPR4-GFP* or *35S:ZjWPR5-GFP* construct. A second injection was performed seven days after the initial one. Following two rounds of infection, leaf and branch samples were collected for RNA extraction, and qRT-PCR was subsequently carried out to confirm target gene overexpression. Those lines with verified overexpression were selected for subsequent experiments.

### 4.8. RNA Extraction and qRT-PCR Analysis

Total RNA was extracted using the Quick Plant RNA Extraction Kit (TianGen Biotechnology, Beijing, China). First-strand cDNA was synthesized from 500 ng of total RNA using the Pre-mix Kit for cDNA Synthesis (TianGen Biotechnology, Beijing, China). RNA quality and concentration were assessed using a NanoDrop 2000 spectrophotometer (Thermo Fisher Scientific, Waltham, MA, USA). All cDNA samples were stored at −20 °C until further use. To quantify the expression of *ZjWPR4* and *ZjWPR5* in jujube leaves, qRT-PCR was performed on a Bio-Rad iQ™5 instrument. The reaction employed TransStart Top Green qPCR SuperMix AQ131 (TransGen Biotech, Beijing, China) in a total volume of 20 µL, composed of 10 µL of 2× SYBR Premix ExTaq™, 0.4 µL each of 10 µM gene-specific primers, 1 µL of template cDNA (diluted), and 8.2 µL of nuclease-free water. After an initial 3 min incubation at 95 °C, the amplification proceeded for 40 cycles: 95 °C for 5 s, 55–62 °C for 15 s, and 72 °C for 15 s. The primers used are as follows: ZjWPR4-F:GGGAGTGAAGTGGGTCGGT, ZjWPR4-R:CTTGGTGTGGGAGGGTGGT. ZjWPR5-F:GGGAAATAAGGGCTGAGAC, ZjWPR5-R:CCCGAATGGAGGGGAAGTA. ZjActin was used as an internal control. ZjActin-F: AGCCTTCCTGCCAACGAG, ZjActin-R: TTGCTTCTCACCCTTGATGC [32]. Each experiment conducted three times independently and data presented as mean ± SD (*n* = 3).

### 4.9. Measurement of Chlorophyll Fluorescence Kinetic Parameters

Freshly collected leaf specimens were placed on the detection stage of a PlantExplorerPro+ (Pheno Vation, Rotterdam, The Netherlands) multipurpose plant photosynthesis imaging system. Appropriate data acquisition modules were selected according to the target parameters. Before measurement, the leaves were adapted to darkness for 30 min to ensure that the Photosystem II (PSII) reaction centers remained fully open and the electron transport chain was in an oxidized state. Upon sudden exposure to visible light, the leaf samples emitted a dark-red fluorescence signal that initially rose and then declined. The starting intensity of this signal was recorded as the minimum fluorescence (Fo), while the peak intensity was recorded as the maximum fluorescence (Fm). The maximum photochemical efficiency of PSII was then derived as Fv/Fm = (Fm − Fo)/Fm.

Subsequently, an actinic light was switched on to mimic the ambient light conditions experienced by the plants. After the fluorescence signal stabilized at a lower level, the steady-state fluorescence (Fs’) was recorded. A second saturating light pulse was then applied to determine the light-adapted maximum fluorescence (Fm′). From these values, the actual photochemical efficiency of PSII was calculated as Fq′/Fm′ = (Fm′ − Fs′)/Fm′, along with the electron transport rate (ETR).

## Figures and Tables

**Table 1:** Analysis of the physicochemical properties of *ZjWPR* gene family proteins.

Gene	Number of Amino Acid	Theoretical pI	Molecular Weight/Da	Atomic Composition	Instability	Aliphatic Index	Hydrophilicity
*ZjWPR1*	898	5.17	98,162.54	C4200H6927N1221O1443S17	57.76	78.23	−0.732
*ZjWPR2*	593	8.22	66,080.25	C2860H4739N831O919S19	42.08	82.38	−0.664
*ZjWPR3*	653	5.48	72,723.01	C3123H5223N901O1057S13	47.78	80.84	−0.806
*ZjWPR4*	582	5.62	65,605.25	C2824H4725N805O950S14	52.35	77.92	−0.871
*ZjWPR5*	594	6.82	66,622.84	C2913H4725N831O910S21	52.28	77.53	−0.721
*ZjWPR6*	622	9.49	70,370.78	C3031H5102N888O972S26	47.97	79.57	−0.775
*ZjWPR7*	574	5.03	65,228.07	C2818H4662N782O937S23	42.11	86.45	−0.749
*ZjWPR8*	490	8.19	55,801.44	C2421H4026N694O787S11	46.02	83.67	−0.862
*ZjWPR9*	213	6.39	23,989.32	C1054H1722N292O334S5	43.87	78.73	−0.744
*ZjWPR10*	338	9.71	38,987.56	C1666H2817N517O532S12	49.82	76.57	−0.98

## Data Availability

The original contributions presented in this study are included in the article. Further inquiries can be directed to the corresponding author.
